# Retraction: Internet-Based Supportive Interventions for Family Caregivers of People With Dementia: Systematic Review and Meta-Analysis

**DOI:** 10.2196/106735

**Published:** 2026-08-20

**Authors:** JMIR Publications Editorial Office

**Affiliations:** 1 JMIR Publications Toronto, ON

The JMIR Publications Editorial Office is retracting the article “Internet-Based Supportive Interventions for Family Caregivers of People With Dementia: Systematic Review and Meta-Analysis” [1] due to concerns regarding the integrity of the peer review process.

Author Minmin Leng responded, indicating that there were undisclosed conflicts of interest with two peer reviewers relating to previous co-publications. The Editorial Office has determined that there is sufficient evidence indicating that the peer review process was compromised to proceed with a retraction.

The authors disagreed with the decision to retract the article.
